# National Association for the Feeble-Minded

**Published:** 1906-11-24

**Authors:** 


### NATIONAL ASSOCIATION FOR THE FEEBLE-MINDED.

The National Association for the Feeble-minded held a conference at Denison House on November 8, which, in spite of the weather, was attended by many of those who feel a deep interest in this peculiarly unfortunate class. Sir William Chance presided. The tone of the whole conference was set in the first paper, by Mrs. Hume Pinsent, on "The Importance of After-care Committees Wherever Special Schools Exist." The whole question of the feeble-minded and what can be done for their welfare depend on this matter of after-care. The institution of special schools has raised many of these unfortunates from wretchedness and made them useful, in some cases self-supporting, members of the community, but inevitably they need a guiding hand all through their life, and without that they are apt to be led into evil. One of the saddest features of the problem of the feeble-minded is the danger to girls of weak mind, who are led astray by unprincipled men, and thereby carry on their weakness to another generation. Another difficulty is the finding of suitable work for feeble-minded boys, but it is felt that this can be met by the establishment of farm colonies, if the funds for these were forthcoming. An appeal for the necessary money was circulated at the conference, the first of the signatories being the Princess Christian. The main point of this appeal, as of the papers of one speaker after another, was the importance of the segregation of the feeble-minded, with a view to preventing the transmission of their weakness to their children. It has been proved that, given sufficient care and supervision, the feeble-minded can do most kinds of ordinary work. They can make boots, clothing, baskets, and mats. They can do printing, carpentering, gardening, and agriculture; and girls are quite fit for laundry and household work, needlework, and weaving. Thus a colony of feeble-minded might become, if once established, very nearly self-supporting, and the very weaknesses of the class—their simplicity, their affectionate nature, their readiness to be guided by others—makes it the more hopeful that when the guidance is kind and wise, they will remain in the paths of wisdom and virtue.

Dr. W. A. Potts dwelt on the need of differentiating between hereditary weakness of intellect and that which is due to special causes, such as accidents, or sending children to school too soon. In dealing with the question, the health of the parents should be investigated where possible. Alcoholism and consumption in the parents may cause weakness of mind in the offspring, and Dr. Potts regarded the employment of married women in factories as a contributing cause. He also wished legislation to step in to prevent the marriage of feeble-minded persons. Dr. Ashby, who followed Dr. Potts, held the same opinions as to the causes of feeble-mindedness, but dwelt strongly on the importance of following out the family histories where the weakness existed, tracing out the careers not only of the immediate relations, but of uncles and aunts and cousins, to find out if it was accidental or hereditary, for, he said, nurture must be taken into account as well as nature. Sir Edward Brabrook spoke of the importance of all after-care associations following uniform methods of investigation, and pointed out that the noting of the few essential facts was more valuable than the collection of a great deal of miscellaneous information. The whole tone of the conference tended to emphasise the need for after-care, and its success may be measured by the fact that at the conclusion several new after-care associations were formed. Reports from several of the existing associations were read, though some of the newer branches preferred to say nothing about their work as yet. Many public bodies profit by the work of the National Association for the Feeble-minded, and some help its work in various ways. But while its educational value is fully recognised, many people seem to underrate or be indifferent to its preventive importance. Thus the representative of one home complains that Boards of Guardians are inclined to remove feeble-minded girls from the home when they find that the training will not succeed in fitting them for service, and taking them back to the workhouse—by no means a suitable abode for creatures so likely to be influenced by stronger wills, and so little capable of judging whether that influence is good or evil. All boards are not so short-sighted, and as the work of the association becomes better known, we are sure that all will co-operate in a work, the final outcome of which must certainly be to lower the rates by preventing the perpetuation of feeble-mindedness from one generation to another.

